# The need for a cardionephrology subspecialty

**DOI:** 10.1093/ckj/sfab054

**Published:** 2021-03-10

**Authors:** Javier Díez, Alberto Ortiz

**Affiliations:** 1 Departments of Nephrology and Cardiology, University of Navarra Clinic, Pamplona, Spain; 2 Program of Cardiovascular Diseases, Center of Applied Medical Research, University of Navarra, Pamplona, Spain; 3 Red de Investigación Renal, Madrid, Spain; 4 Division of Nephrology IIS-Fundación Jiménez Díaz, University Autonoma of Madrid, Madrid, Spain

**Keywords:** cardionephrology, cardiovascular disease, chronic kidney disease

## Abstract

Chronic kidney disease (CKD) has structural and functional repercussions for the cardiovascular system that facilitate the development of cardiovascular disease (CVD). In fact, cardiovascular complications are frequent in the CKD population and thus cause a great clinical, public health and economic burden. Despite this challenge, the prevention and management of cardiovascular complications is one among several aspects of CKD that meets the criteria of an unmet medical need. This probably has to do with the misperception by the nephrologist of the global relevance of CVD in the CKD patient which, in turn, may be due to insufficient cardiovascular training during nephrology specialization. Therefore a change in approach is necessary to understand CKD as a disease in which the manifestations and complications related to CVD become so frequent and important that they require dedicated multidisciplinary clinical management. From this perspective, it makes sense to consider training in the subspecialty of cardionephrology to provide adequate cardiovascular care for CKD patients by the nephrologist. In addition, the cardionephrology subspecialist would be better able to interact with other specialists in multidisciplinary care settings created to achieve a deeper understanding and more effective clinical handling of the interactions between CKD and CVD.

As recommended 20 years ago by the American Heart Association (AHA) [1] and recognized in the current European guidelines on cardiovascular disease (CVD) prevention [2], people with chronic kidney disease (CKD) should be considered in the highest-risk group for the prevention, detection and treatment of CVD. However, management of CVD in CKD patients is still an unmet medical need [3, 4]. Among the various reasons that may explain this inconsistency is the lack of global perception of CVD in the CKD patient by the nephrologist. In fact, although CKD is now viewed as a systemic and multiorgan disease [5, 6], its cardiovascular aspects do not receive the attention that corresponds to their burden of disease and the nephrologist’s intervention is limited to cardiovascular risk reduction (e.g. blood lipids and blood pressure management). Therefore a new approach towards CKD as an initially renal disease that evolves to become predominantly a CVD is required. And, as a consequence of this, it is also necessary to rethink the training of specialists in nephrology to give more relevance to specific knowledge and practice in cardiovascular medicine [7, 8].

The aim of this editorial is to emphasize the most relevant epidemiological, pathophysiological and clinical evidence that supports the view of CVD in CKD patients as a challenge with implications for the nephrologist, in the sense that its proper management should be the subject of the subspeciality of cardionephrology integrated in multidisciplinary cardiorenal teams.

## THE CHALLENGE OF CVD IN CKD PATIENTS

### CVD enhances the medical, health and economic burden of CKD

**FIGURE 1:**
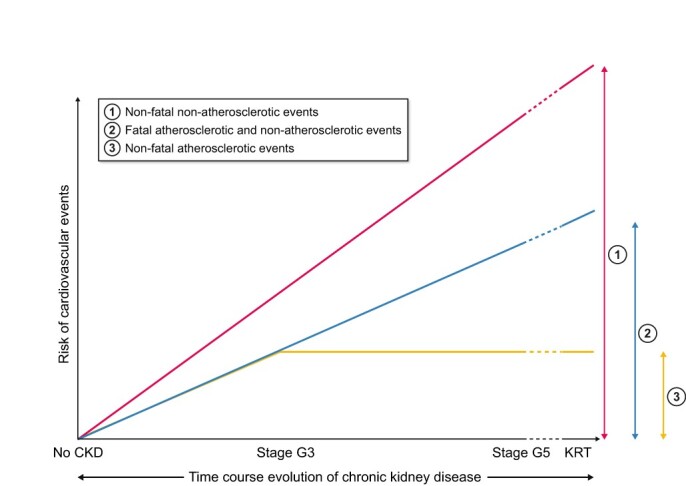
Conceptual arbitrary representation of the risk of cardiovascular events along the progression of CKD (independent of the time that a patient spends in each stage of the Kidney Disease: Improving Global Outcomes classification) and during KRT (independent of the modality of therapy). Partially adapted from Wanner C, Amman K, Shoji T. *Lancet* 2016; 388: 276–284.

As glomerular filtration rate (GFR) declines and/or the urinary loss of albumin increases and CKD progresses, so does the incidence and prevalence of CVD and its complications [9, 10] (Figure 1). Systematic analyses of the world burden of CKD reveal that it has a major effect on global health, both as a direct cause of global morbidity and mortality and as an important risk factor for CVD-mediated morbidity and mortality [11, 12]. In this regard, it is increasingly apparent that individuals with CKD are more likely to die of CVD than to develop kidney failure [11] or to die from kidney failure [11]. In addition, CVD is less well tolerated in patients with CKD than in patients without CKD. For instance, heart failure (HF) hospitalizations and cardiovascular mortality are higher in HF patients with CKD than in patients without CKD [13]. On the other hand, patients with CKD are at high risk for acute kidney injury (AKI) and accumulating evidence supports the notion that cardiovascular damage due to AKI increases both short-term and long-term cardiovascular risk in CKD [14].

Data from the US Renal Data System show the enhancer effect of cardiovascular complications on the economic burden of CKD, thus aggravating the tremendous impact of CKD on healthcare budgets [15]. In particular, the high rates of hospitalization due to cardiovascular complications increase the direct costs, thus increasing the economic burden of CKD from its early stages [16].

### CVD is facilitated by the impact of CKD on blood vessels and the heart

CKD facilitates the development of systemic macro- and microvascular damage, as well as cardiac remodelling, through multiple pathways. Indeed, several studies have shown associations between CKD and atherosclerosis-related clinical complications [17] and subclinical atherosclerosis prevalence and progression [18]. The prevalence of calcifications in the intima and media of the arterial wall is increased in CKD patients, likely as a result of complex interactions between atherosclerotic changes in the vascular bed, mineral metabolites and other uremic factors [19], and is associated with adverse cardiovascular outcomes. For instance, coronary calcification is associated with myocardial infarction, HF and all-cause mortality in CKD patients [20]. Finally, studies evaluating the structure and function of the microcirculation in humans [21] and animals [22] with CKD reported that capillary rarefaction resulting in decreased microvascular density is a constant finding in different organs, including the heart.

In accordance with the US Renal Data System, the incidence and prevalence of non-cardiac diseases different from atherosclerotic ischaemic heart disease (e.g., cardiomyopathies and valvular diseases) is higher in CKD patients than in non-CKD patients, irrespective of the influence of non-kidney-related potential confounding factors [15]. Of note, cardiac diseases in CKD patients are characterized by progression to HF [23]. Recent experimental and clinical evidence suggests that fibrosis of the myocardium may facilitate the development of HF in CKD patients, as several mechanisms are operative along the different stages of CKD that may converge to alter fibroblasts and collagen turnover in the heart, thus resulting in myocardial remodelling that alters both diastolic and systolic function [24].

### Clinical management of CVD is suboptimal in patients with later stages of CKD

The high incidence of cardiovascular events in patients with kidney failure and patients on kidney replacement therapy (KRT) (Figure 1) could also be related to the fact that these populations are less likely to be diagnosed with CVD and to receive appropriate cardiovascular protective treatments. Indeed, CVD is usually underdiagnosed in these patients, as cardiovascular protocols are underrepresented in the general screening and diagnostic procedures for CKD [25]. In addition, the sensitivities and specificities of both the clinical manifestations and imaging and biochemical cardiovascular tests are questionable in patients with later stages of CKD, as it is the case for the diagnosis of HF [26]. On the other hand, patients with kidney failure, and particularly patients on KRT, are usually excluded from cardiovascular clinical trials conducted in the general population or in at-risk populations [27, 28]. Thus, as evidence-based management of CVD is lacking, the efficacy and safety of cardiovascular therapies in these patients are uncertain [29]. The underuse of diverse therapeutic alternatives with cardiovascular impact (within a wider attitude of therapeutic nihilism) has repeatedly been associated with adverse outcomes in patients with kidney failure [30]. However, this is also observed earlier in the course of CKD, especially for older individuals [31].

An additional aspect to consider in the challenging management of CVD in patients on KRT is the detrimental cardiovascular effect of dialysis, specially haemodialysis [15], and to a lesser extent of kidney transplantation [32], which would explain the high cardiovascular risk of patients on KRT (Figure 1). Systemic circulatory ‘stress’ resulting from haemodialysis and acting as a CVD amplifier [33] and the cardiometabolic effects of immunosuppressive therapies in kidney transplanted patients [34] may account for additional cardiovascular risk in these populations. In this regard, the European Society of Cardiology Systemic Coronary Risk Evaluation (ESC-SCORE), Framingham Heart Study SCORE (Framingham-SCORE), Prospective Cardiovascular Munster Study SCORE (PROCAM-SCORE) or Assessing cardiovascular risk using Scottish Intercollegiate Guidelines Network SCORE (ASSIGN-SCORE) algorithms can predict cardiovascular risk after kidney transplantation at the time of entering the waiting list and may be used to guide therapy [35].

## IMPLICATIONS OF THE CHALLENGE FOR THE NEPHROLOGIST

The relevance of CVD in patients with CKD is a real challenge for the nephrologist that can only be faced through a demanding training plan and an appropriate clinical environment. The first must be the objective of the subspecialty of cardionephrology and the second must be the result of collaboration with other specialists in cardiorenal care teams.

Current general nephrology training does not seem to be sufficient for covering the broad and rapidly evolving field of cardiovascular medicine and thus a specific training programme to enhance the knowledge and clinical capability of the nephrologist in CVD is needed [7]. A variety of topics related to acute and chronic clinical cardiac and vascular problems present in CKD patients must be considered for inclusion in the programme [8]. The cardionephrology training programme may be offered both as a cumulative period during the nephrology specialization time and as a subspecialty track to allow senior nephrologists the opportunity to train and develop proficiency in cardiovascular medicine [36].

The subspecialty of cardionephrology offers the nephrologist the opportunity to integrate into dedicated cardiorenal interdisciplinary teams jointly involved in early identification and appropriate management of patients with the dual burden of CKD and CVD across the inpatient and outpatient settings [37]. These cardiorenal multidisciplinary teams represent an optimal setting for the development of clinical studies aimed at increasing evidence-based practice in the diagnosis and treatment of CVD in patients with kidney failure and patients on KRT [38].

Finally, the implementation of the subspecialty of cardionephrology in daily clinical practice can be an opportunity contributing to broadening the academic and scientific horizons of nephrology in collaboration with other specialties, as well as stimulating clinical trials research, an area where nephrology has fallen greatly behind other specialties [39]. In addition, the subspecialty of cardionephrology may facilitate the recruitment of the new generations of nephrologists [40, 41], thus helping to attract trainee physicians [42].

In summary, it is necessary to recognize that the development of CVD in patients with CKD has such important clinical and health consequences that it challenges the capacities of current nephrologists to deal with them. Therefore, the time has come to encourage and support the training of subspecialists in cardionephrology with the clear objective of minimizing the burden that CVD places on CKD. In this framework, the authors of this article call on the members of the Union of European Medical Specialties Renal Section and the European Renal Association–European Dialysis and Transplant Association responsible for the European Certificate in Nephrology (ECN) [43] to consider knowledge and handling of cardiorenal issues among the most demanding requirements of the ECN.

## CONFLICT OF INTEREST STATEMENT

J.D. has received consultancy or speaker fees or travel support from Bayer and AstraZeneca. A.O. has received consultancy or speaker fees or travel support from AstraZeneca, Amicus, Amgen, Fresenius Medical Care, Bayer, Sanofi-Genzyme, Menarini, Kyowa Kirin, Alexion, Otsuka and Vifor Fresenius Medical Care Renal Pharma and is Director of the Catedra Mundipharma–UAM of diabetic kidney disease and the Catedra AstraZeneca–UAM of chronic kidney disease and electrolytes. This article has not been published previously in whole or part.

## References

[1] Sarnak MJ , Levey AS, Schoolwerth AC et al Kidney disease as a risk factor for development of cardiovascular disease: a statement from the American Heart Association Councils on Kidney in Cardiovascular Disease, High Blood Pressure Research, Clinical Cardiology, and Epidemiology and Prevention. Circulation 2003; 108: 2154–2169 14581387 10.1161/01.CIR.0000095676.90936.80

[2] Piepoli MF , Hoes AW, Agewall S et al 2016 European guidelines on cardiovascular disease prevention in clinical practice. The Sixth Joint Task Force of the European Society of Cardiology and Other Societies on Cardiovascular Disease Prevention in Clinical Practice (constituted by representatives of 10 societies and by invited experts). Developed with the special contribution of the European Association for Cardiovascular Prevention & Rehabilitation (EACPR). Eur Heart J 2016; 37: 2315–2381 27222591 10.1093/eurheartj/ehw106 PMC4986030

[3] Bello AK , Alrukhaimi M, Ashuntantang GE et al Complications of chronic kidney disease: current state, knowledge gaps, and strategy for action. Kidney Int Suppl (2011) 2017; 7: 122–129 30675426 10.1016/j.kisu.2017.07.007 PMC6341007

[4] Levin A , Tonelli M, Bonventre J et al Global kidney health 2017 and beyond: a roadmap for closing gaps in care, research, and policy. Lancet 2017; 390: 1888–1917 28434650 10.1016/S0140-6736(17)30788-2

[5] Romagnani P , Remuzzi G, Glassock R et al Chronic kidney disease. Nat Rev Dis Primers 2017; 3: 17088 29168475 10.1038/nrdp.2017.88

[6] Zoccali C , Vanholder R, Massy ZA et al The systemic nature of CKD. Nat Rev Nephrol 2017; 13: 344–358 28435157 10.1038/nrneph.2017.52

[7] Ronco C , Ronco F, McCullough PA. A call to action to develop integrated curricula in cardiorenal medicine. Blood Purif 2017; 44: 251–259 29065398 10.1159/000480318

[8] Kazory A , McCullough PA, Rangaswami J et al Cardionephrology: proposal for a futuristic educational approach to a contemporary need. Cardiorenal Med 2018; 8: 296–301 30089281 10.1159/000490744 PMC6477482

[9] Foley RN , Parfrey PS, Sarnak MJ. Clinical epidemiology of cardiovascular disease in chronic renal disease. Am J Kidney Dis 1998; 32(5 Suppl 3): S112–S119 9820470 10.1053/ajkd.1998.v32.pm9820470

[10] Matsushita K , van der Velde M, Astor BC et al Association of estimated glomerular filtration rate and albuminuria with all-cause and cardiovascular mortality in general population cohorts: a collaborative metaanalysis. Lancet 2010; 375: 2073–2081 20483451 10.1016/S0140-6736(10)60674-5 PMC3993088

[11] Thomas B , Matsushita K, Abate KH et al Global cardiovascular and renal outcomes of reduced GFR. J Am Soc Nephrol 2017; 28: 2167–2179 28408440 10.1681/ASN.2016050562 PMC5491277

[12] GBD Chronic Kidney Disease Collaboration. Global, regional, and national burden of chronic kidney disease, 1990–2017: a systematic analysis for the Global Burden of Disease Study 2017. Lancet 2020; 395: 709–733 32061315 10.1016/S0140-6736(20)30045-3 PMC7049905

[13] Galil AG , Pinheiro HS, Chaoubah A et al Chronic kidney disease increases cardiovascular unfavourable outcomes in outpatients with heart failure. BMC Nephrol 2009; 10: 31 19843342 10.1186/1471-2369-10-31 PMC2771010

[14] Legrand N , Rossignol P. Cardiovascular consequences of acute kidney injury. N Engl J Med 2020; 382: 2238–2247 32492305 10.1056/NEJMra1916393

[15] US Renal Data System 2018 Annual Data Report. Am J Kidney Dis 2019; 73(3 Suppl 1): S1–S772 10.1053/j.ajkd.2019.01.001 PMC6620109 30798791

[16] Wang V , Vilme H, Maciejewski ML et al The economic burden of chronic kidney disease and end-stage renal disease. Semin Nephrol 2016; 36: 319–330 27475662 10.1016/j.semnephrol.2016.05.008

[17] Keith DS , Nichols GA, Gullion CM et al Longitudinal follow-up and outcomes among a population with chronic kidney disease in a large managed care organization. Arch Intern Med 2004; 164: 659–663 15037495 10.1001/archinte.164.6.659

[18] Betriú A , Martínez-Alonso M, Arcidiacono MV et al Prevalence of subclinical atheromatosis and associated risk factors in chronic kidney disease: the NEFRONA study. Nephrol Dial Transplant 2014; 29: 1415–1422 24586070 10.1093/ndt/gfu038

[19] Disthabanchong S , Srisuwarn P. Mechanisms of vascular calcification in kidney disease. Adv Chronic Kidney Dis 2019; 26: 417–426 31831120 10.1053/j.ackd.2019.08.014

[20] Chen J , Budoff MJ, Reilly MP et al Coronary artery calcification and risk of cardiovascular disease and death among patients with chronic kidney disease. JAMA Cardiol 2017; 2: 635–643 28329057 10.1001/jamacardio.2017.0363 PMC5798875

[21] Amann K , Törnig J, Buzello M et al Effect of antioxidant therapy with dl-α-tocopherol on cardiovascular structure in experimental renal failure. Kidney Int 2002; 62: 877–884 12164869 10.1046/j.1523-1755.2002.00518.x

[22] Charytan DM , Padera R, Helfand AM et al Increased concentration of circulating angiogenesis and nitric oxide inhibitors induces endothelial to mesenchymal transition and myocardial fibrosis in patients with chronic kidney disease. Int J Cardiol 2014; 176: 99–109 25049013 10.1016/j.ijcard.2014.06.062 PMC4161362

[23] House AA , Wanner C, Sarnak MJ et al Heart failure in chronic kidney disease: conclusions from a Kidney Disease: Improving Global Outcomes (KDIGO) Controversies Conference. Kidney Int 2019; 95: 1304–1317 31053387 10.1016/j.kint.2019.02.022

[24] Romero-González G , González A, López B et al Heart failure in chronic kidney disease: the emerging role of myocardial fibrosis. Nephrol Dial Transplant 2020; doi: 10.1093/ndt/gfaa284 10.1093/ndt/gfaa284 33313766

[25] Chen TK , Knicely DH, Grams ME. Chronic kidney disease diagnosis and management: a review. JAMA 2019; 322: 1294–1304 31573641 10.1001/jama.2019.14745 PMC7015670

[26] Chawla LS , Herzog CA, Costanzo MR et al Proposal for a functional classification system of heart failure in patients with end-stage renal disease: proceedings of the Acute Dialysis Quality Initiative (ADQI) XI workgroup. J Am Coll Cardiol 2014; 63: 1246–1252 24530671 10.1016/j.jacc.2014.01.020

[27] Maini R , Wong DB, Addison D et al Persistent underrepresentation of kidney disease in randomized, controlled trials of cardiovascular disease in the contemporary era. J Am Soc Nephrol 2018; 29: 2782–2786 30389726 10.1681/ASN.2018070674 PMC6287860

[28] Carpenter MA , Weir MR, Adey DB et al Inadequacy of cardiovascular risk factor management in chronic kidney transplantation—evidence from the FAVORIT study. Clin Transplant 2012; 26: E438–E446 22775763 10.1111/j.1399-0012.2012.01676.x PMC4388027

[29] Ruiz-Hurtado G , Sarafidis P, Fernandez-Alfonso MS et al Global cardiovascular protection in chronic kidney disease. Nat Rev Cardiol 2016; 13: 603–608 27053454 10.1038/nrcardio.2016.48

[30] Ortiz A , Covic A, Fliser D et al Epidemiology, contributors to, and clinical trials of mortality risk in chronic kidney failure. Lancet 2014; 383: 1831–1843 24856028 10.1016/S0140-6736(14)60384-6

[31] Villain C , Liabeuf S, Metzger M et al Impact of age on cardiovascular drug use in patients with chronic kidney disease. Clin Kidney J 2020; 13: 199–207 32296525 10.1093/ckj/sfz063 PMC7147308

[32] Ojo AO , Hanson JA, Wolfe RA et al Long-term survival in renal transplant recipients with graft function. Kidney Int 2000; 57: 307–313 10620213 10.1046/j.1523-1755.2000.00816.x

[33] Canaud B , Kooman JP, Selby NM et al Dialysis-induced cardiovascular and multiorgan morbidity. Kidney Int Rep 2020; 5: 1856–1869 33163709 10.1016/j.ekir.2020.08.031 PMC7609914

[34] Rangaswami J , Mathew RO, Parasuraman R et al Cardiovascular disease in the kidney transplant recipient: epidemiology, diagnosis and management strategies. Nephrol Dial Transplant 2019; 34: 760–773 30984976 10.1093/ndt/gfz053

[35] Herzog AL , Kalogirou C, Wanner C et al Comparison of different algorithms for the assessment of cardiovascular risk after kidney transplantation by the time of entering waiting list. Clin Kidney J 2020; 13: 150–158 32296518 10.1093/ckj/sfz041 PMC7147301

[36] Sachdeva M , Shah AD, Singh HK et al Opportunities for subspecialization in nephrology. Adv Chronic Kidney Dis 2020; 27: 320–327 33131645 10.1053/j.ackd.2020.05.002

[37] Rangaswami J , Bhalla V, Blair JEA et al Cardiorenal syndrome: classification, pathophysiology, diagnosis, and treatment strategies: a scientific statement from the American Heart Association. Circulation 2019; 139: e840–e878 30852913 10.1161/CIR.0000000000000664

[38] Baigent C , Herrington WG, Coresh J et al Challenges in conducting clinical trials in nephrology: conclusions from a Kidney Disease–Improving Global Outcomes (KDIGO) Controversies Conference. Kidney Int 2017; 92: 297–305 28709600 10.1016/j.kint.2017.04.019 PMC6326036

[39] Inrig JK , Califf RM, Tasneem A et al The landscape of clinical trials in nephrology: a systematic review of Clinicaltrials.gov. Am J Kidney Dis 2014; 63: 771–780 24315119 10.1053/j.ajkd.2013.10.043 PMC3988265

[40] Rangaswami J , Mathew RO, McCullough PA. Resuscitation for the specialty of nephrology: is cardionephrology the answer? Kidney Int 2018; 93: 25–26 29137816 10.1016/j.kint.2017.10.002

[41] Rondeau E , Luyckx VA, Anders HJ et al Challenges and opportunities for nephrology in Western Europe. Kidney Int 2019; 95: 1037–1040. 30777285 10.1016/j.kint.2018.09.028

[42] Bello A , Levin A, Lunney M et al ISN Global Kidney Health Atlas 2019. Brussels: International Society of Nephrology, 2019. https://www.theisn.org/focus/ckd#health-atlas

[43] Cannata-Andía JB , Weinstein T, Slotki I et al The European Certificate in Nephrology: towards harmonization and excellence in training. Clin Kidney J 2019; 12: 167–171 30976392 10.1093/ckj/sfy106 PMC6452178

